# Innovative Technologies: X-Rays Get in Synch

**Published:** 2006-01

**Authors:** Graeme Stemp-Morlock

Synchrotrons may have been designed with high-energy physics in mind, but now biologists are starting to see the light too. Jeffrey Gillow, a researcher at Brookhaven National Laboratory, has been making use of the X-ray microscope at the National Synchrotron Light Source (NSLS) in New York to see extremely fine details of bacteria biochemistry in a technique known as X-ray spectromicroscopy.

Gillow’s team, funded by the Department of Energy Office of Science, uses “soft” X-rays (up to 800 electronvolts, a relatively small amount of energy) to study the chemical structure of organic compounds. “It’s great because you get more than just a detailed picture,” says Gillow. “You also get chemical information about your sample.”

Gillow uses the synchrotron to precisely tune the energy of the X-rays, knocking carbon electrons out of their orbitals. The resulting disturbance changes the bonds of molecules, and the researchers can read the spectra to see which elements were bonded to which.

The precise nature of the X-ray microscope allows Gillow to see exacting chemical detail within bacteria. Recently, his team used the 30-nanometer resolution of the NSLS X-ray microscope to observe an immature spore develop within a *Clostridium* sp. bacterium, something far too minute and hidden within its host for any conventional electron microscope. These findings were published in the June 2005 issue of the *Journal of Electron Spectroscopy and Related Phenomena*.

Another strength of X-ray spectromicroscopy is that samples require only minimal preparation. Says Gillow, “There is no staining necessary. Basically you just put the sample on the window and away you go.” Without staining or heat fixing, the bacterium maintains its naturally occurring biochemical composition.

However, X-ray spectromicroscopy does require that experiments be conducted in close proximity to a synchrotron. And even though there are currently 40 of these very expensive machines in the world, only a few have the capabilities to conduct this type of research. Further, no live specimens can be studied due to the extraordinary amount of radiation they receive.

Regardless, X-ray spectromicroscopy offers environmental scientists chemical detail and unaltered observations like never before, which is key to understanding the complex biochemical reactions that bacteria undergo in the environment. For example, groups interested in bioremediation can now see on a molecular scale how bacteria alter the chemistry of metals and radionuclides and remove them from soils and waters.

A better understanding of subcellular microorganism chemistry, specifically sporulation, might also help authorities neutralize bioterrorism threats before they become a problem. “Finding ways to interrupt sporulation could stop bioterrorism attacks,” says Gillow. “But I doubt you will ever see a synchrotron at an airport scanning your luggage.”

